# Cu-doped polypyrrole hydrogel with tumor catalyst activity for NIR-II thermo-radiotherapy

**DOI:** 10.3389/fbioe.2023.1225937

**Published:** 2023-07-07

**Authors:** Shile Wang, Haotian Fei, Yuhong Ma, Daoming Zhu, Hongtao Zhang, Xiang Li, Qinqin Huang

**Affiliations:** ^1^ The Research and Application Center of Precision Medicine, The Second Affiliated Hospital of Zhengzhou University, Zhengzhou, China; ^2^ Department of Pharmacy/Evidence-Based Pharmacy Center, West China Second University Hospital, Sichuan University, Chengdu, Sichuan, China; ^3^ Department of Psychiatry, Huaian No. 3 People’s Hospital, Huai’an, Jiangsu, China; ^4^ Department of Electronic Science and Technology, School of Physics and Technology, Wuhan University, Wuhan, China; ^5^ Blood Purification Center, The People’s Hospital of Zhengzhou University, Zhengzhou, China; ^6^ Blood Purification Center, Henan Provincial People’s Hospital, Zhengzhou, China; ^7^ Department of Central Laboratory and Precision Medicine Center, The Affiliated Huai’an Hospital of Xuzhou Medical University and Huai’an Second People’s Hospital, Huai’an, China; ^8^ Department of Nephrology, The Affiliated Huai’an Hospital of Xuzhou Medical University and Huai’an Second People’s Hospital, Huai’an, China

**Keywords:** radiotherapy, nanozyme, hydrogel, photothermal therapy, ROS

## Abstract

**Introduction:** Radiotherapy (RT) is one of the key methods for treating breast cancer. However, the effect of single RT is often poor because of insufficient deposition of X-rays in tumor sites and radiation resistance induced by the abnormal tumor microenvironment (overexpression of glutathione (GSH)). The development of multifunctional RT sensitizers and synergetic therapeutic strategies is, therefore, a promising area for enhancing the anticancer effect of RT.

**Methods:** In this study, a multifunctional nanozyme hydrogel based on Cu-doped polypyrrole (CuP) was designed to work concertedly with a second near-infrared thermal RT. The CuP-based hydrogel (CH) reached the tumor site when injected *in-situ* and achieved long-term storage.

**Results:** Once stimulated with 1064-nm laser irradiation, the heated and softened hydrogel system released CuP nanozyme to provide photothermal therapy, thereby inhibiting the repair of DNA damage caused by RT. In addition, CuP with dual nanozyme activity depleted the intracellular GSH to reduce the antioxidant capacity of the tumor. Moreover, CuP converted H_2_O_2_ to produce ·OH to directly kill the tumor cells, thus enhancing the capability of low-dose RT to inhibit tumor growth. *In vivo* experiments showed that the CH system used in combination with a low-power 1064-nm laser and low-dose RT (4 Gy) exhibited good synergistic anticancer effects and biological safety.

**Discussion:** As a new light-responsive hydrogel system, CH holds immense potential for radio-sensitization.

## Introduction

Cancer is one of the most life-threatening diseases in the world (Li et al., 2022). Approximately 70% of patients with cancer need radiotherapy (RT) (Limoli and Vozenin, 2023; Lin et al., 2023; Wu et al., 2023). RT mainly uses high-energy X-rays or *γ*-rays to induce DNA damage and cell apoptosis, thus resulting in tumor ablation (Secchi et al., 2023). When an adequate dose of radiation is used to locally irradiate the cancer tissue, it fights against cancer cells and effectively combats the disease. However, high-dose radiation can also cause damage to the normal cells in the vicinity, which is a concern in the development of RT (Ni et al., 2018; Gao et al., 2022). With the advancements in nanomedicine, several new cancer treatments, such as photothermal therapy (PTT), photodynamic therapy (PDT), and gene therapy, have emerged (Wang et al., 2022a; Wang et al., 2022b; Sun et al., 2022; Zhao et al., 2022). Of these, PTT is a kind of treatment method that uses a photothermal agent to convert light energy into heat energy under the irradiation of external light sources, such as near-infrared (NIR) laser (Ye et al., 2019; Zhu Y. et al., 2022). This irradiation increases the temperature of the tumor site, thus ablating the tumor. Owing to its high selectivity, low invasion, and high efficiency, PTT has been employed widely (Zhu et al., 2023a). The heat generated during PTT not only directly kills tumor cells but also acts as an RT sensitizer. First, hyperthermia induces double-strand breaks in the DNA, thus leading to protein aggregation and inhibiting the repair of DNA damage induced by ion radiation (Wang et al., 2021). Second, the high temperature kills S-phase cells that display the lowest sensitivity to radiation (Lyu et al., 2021). Third, the mild photothermal effect accelerates the blood flow in the tumor, thus increasing the oxygenation in the hypoxic part of the tumor and improving the sensitivity of tumor cells to RT (Lyu et al., 2019). Therefore, the concerted use of PTT/RT has good prospects.

The antitumor effect of PTT/RT is limited to a great extent by the complexity of the tumor physiological environment (poor permeability of the radiosensitizer in RT) and its limitation (limited conversion efficiency of the photothermal agent) (Yin et al., 2017; Cheng et al., 2021; Lan et al., 2021). Owing to the rapid growth and metabolism of tumor cells, the tumor microenvironment (TME) tends to be acidic, with low oxygen and elevated H_2_O_2_ levels (Ren et al., 2020; Zhu D. et al., 2022). Based on these characteristics, a reasonably designed nanozyme system can generate toxic substances in tumors via endogenous properties or exogenous stimulation to kill tumor cells, thereby improving the effects of thermal RT on tumors (Ji et al., 2021; Jiang et al., 2022). In recent years, nanomaterials have gained widespread attention in the field of tumor therapy (Ye et al., 2019; Ren et al., 2020; Sun et al., 2021; Zhao et al., 2022; Chen et al., 2023). The physicochemical response characteristics of nanomaterials are combined with the catalytic activity of enzymes, such as glutathione oxidase (GSH-OXD) and peroxidase (POD) (Zhu Y. et al., 2021; Zhu et al., 2023a; Zhu et al., 2023b). Hence, the development of multifunctional nanozymes can provide a promising weapon for enhancing tumor treatment. Intracellular GSH can offer protection against radiation, including free radical removal, peroxide reduction, and protein mercaptan, to maintain the reduced state (Chang et al., 2020). Emerging studies have shown that mixed-valence copper-based nanomaterials can specifically catalyze the conversion of GSH into glutathione disulfide in tumors in response to the TME, thereby restoring the sensitivity of cells to X-rays. In addition, a Cu^+^-mediated pod-like reaction converts H_2_O_2_ into highly toxic OH to destroy mitochondria, which makes cells more vulnerable to radiation attack (Zhang et al., 2011).

Photothermal agents are generally injected intravenously to deliver them to tumor tissues, and although the biocompatibility and targeting of most photothermal agents can be improved via modification, their efficacy remains unsatisfactory (Dai et al., 2022; Zhuang et al., 2022). Furthermore, the systemic toxicity of nanomaterials, which often leads to unavoidable side effects, should be considered (Xu et al., 2022). Hydrogel is a three-dimensional network polymer that has been widely used in the biomedical field. The functions of the hydrogel include sustained drug release, cell delivery vehicle, and tissue engineering (Hou et al., 2018). Injectable hydrogels with injectable and *in situ* gelling properties are of great interest owing to their biocompatibility, ease of handling, and non-invasive mode of administration via injection (Wu et al., 2019). Such hydrogels exhibit properties that are superior to those of preformed hydrogels. The hydrogel carrying the cargo can prolong the sustained release time of the cargo, immensely reduce the toxicity and systemic side effects, centralize the administration concentration, and augment the utilization rate of the drug (Zhu D. et al., 2021; Zhang et al., 2022). Injectable hydrogels are clinically appealing because they significantly reduce patient discomfort, risk of infection, recovery time, and treatment costs (Chen et al., 2020). For example, Zhang designed light-responsive black phosphorus (BP)-based hydrogel to achieve controlled release of the BP and enable safe photothermal therapy (Qiu et al., 2018). Therefore, light-responsive hydrogels are expected to become the preferred medium for controlled-release nanomaterials.

**SCHEME 1:**
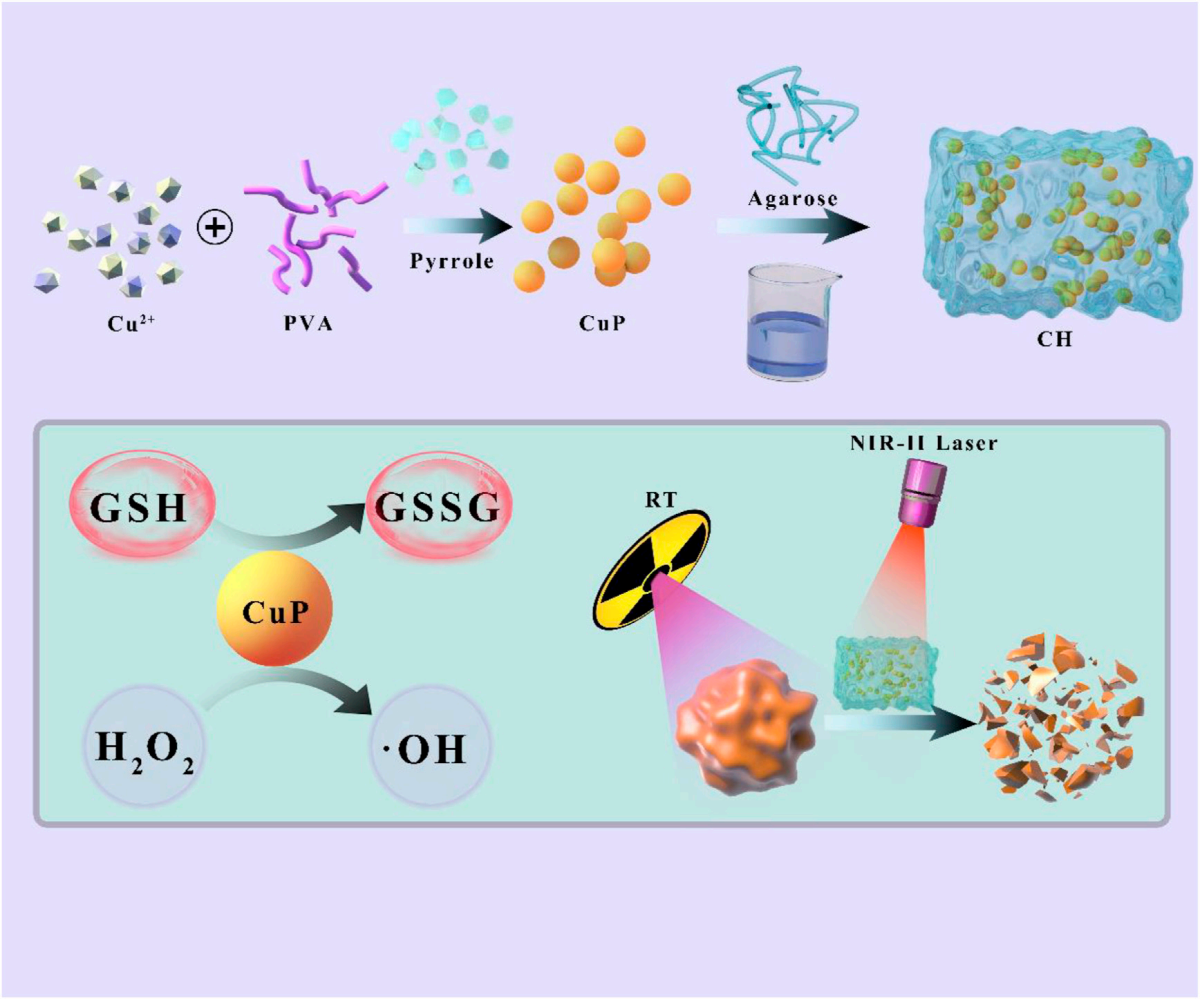
Schematic illustration of Cu-Doped polypyrrole hydrogel with tumor catalyst activity for NIR-II thermo-radiotherapy.

In this study, Cu-doped polypyrrole (CuP), a copper-based multifunctional nanozyme was synthesized and loaded into agarose hydrogel to form a CuP-based hydrogel (CH) to achieve sensitization treatment for breast cancer using a second near-infrared (NIR-II) photothermal synergistic RT (Scheme 1). CH reached the focal area directly when injected intratumorally and accumulated *in situ* after gelation. After irradiation with 1064-nm laser (0.5 W/cm^2^), CH converted light energy into heat energy, thereby enabling accurate and deep penetration of NIR-II PTT. Moreover, the thermally responsive hydrogel gradually softened and released the CuP nanozyme. CuP reduced GSH in the TME and converted H_2_O_2_ to generate highly toxic OH, destroy the redox steady state, and greatly enhance the effect of subsequent RT. Both *in vitro* cell experiments and *in vivo* animal model experiments showed that the designed CH system achieved satisfactory synergistic therapeutic effects. Furthermore, the combination of a relatively low radiation dose (only 4Gy) and a laser power of 0.5 W/cm^2^ resulted in almost no adverse effects during the treatment cycle. This finding confirmed the long-term reliability of the CH system and provided novel insights for the multiple sensitization study of RT for 4T1 tumors.

## Results and discussion

**FIGURE 1:**
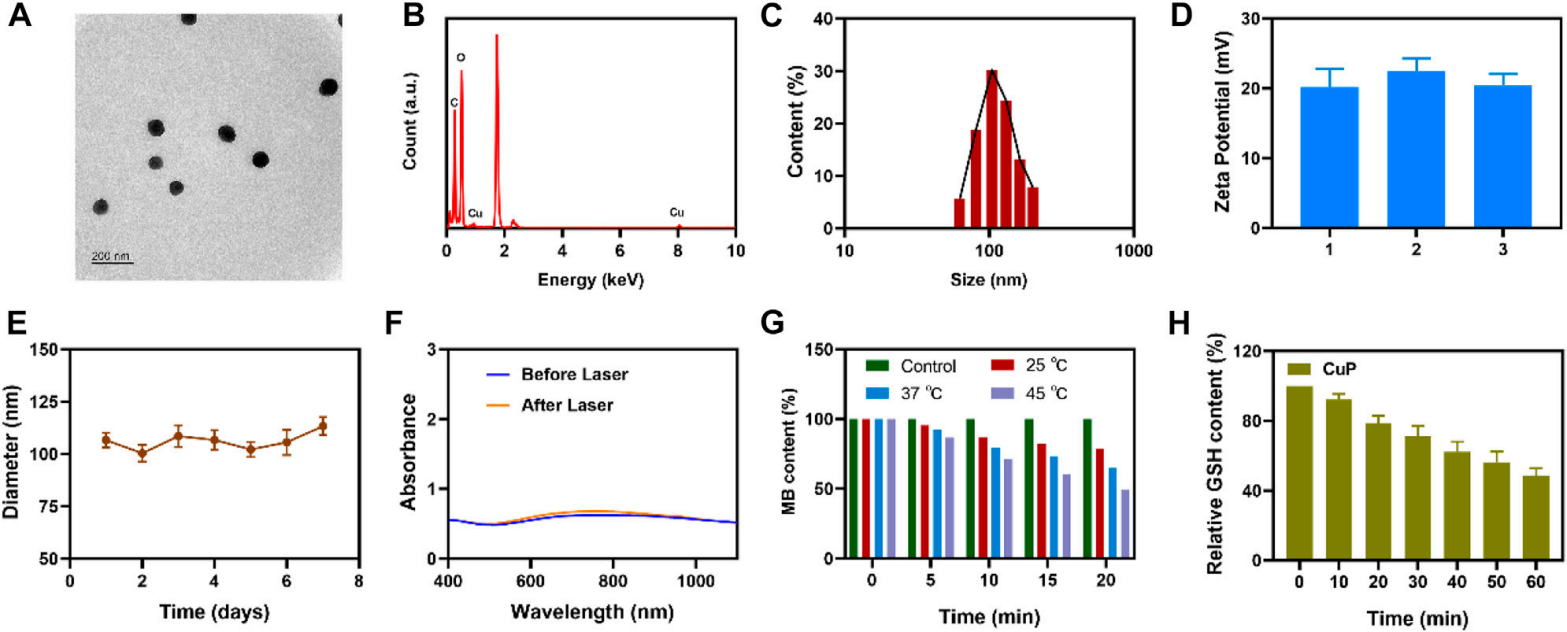
**(A)** TEM image of CuP **(B)** Energy dispersive spectroscopy (EDS) of CuP **(C)** Hydrodynamic diameter of CuP in water **(D)** The zeta potential of CuP suspended in PBS **(E)** DLS diameter of CuP within 7 days **(F)** UV-vis-NIR absorbance spectra of CuP aqueous dispersions before and after 1,064 nm laser irradiation for 20 min **(G)** MB depletion profile treated with or without CuP at different temperature (RT, 37°C, and 45°C) **(H)** The relative GSH content of the supernatant after the reaction of GSH and CuP.

In this study, CuCl_2_ was used as an oxidation catalyst instead of FeCl_3_. *In situ* chemical oxidative polymerization was used to trigger the polymerization of pyrrole monomer at room temperature, and polyvinyl alcohol (PVA) was used as a stabilizer to avoid the infinite growth of CuP. As shown in Figure 1A, transmission electron microscopy (TEM) revealed that the synthesized CuP exhibited good dispersion, uniform size, and spherical characteristics. When the elemental mapping images were analyzed, the major elements Cu, C, and N were distributed (Figure 1B). The size of the CuP nanozyme was controlled to approximately 100 nm by improving the PVA content (Figure 1C). The stability of nanocomposites in solution is critical to achieving a good therapeutic effect (Su et al., 2021; Yang et al., 2022). The zeta potential of the CuP obtained by repeated preparation is almost unchanged (Figure 1D). Hence, the average hydrodynamic diameter change of CuP dispersed in PBS was measured for 1 week using dynamic light scattering (DLS). Figure 1E presents the hydrodynamic diameter of CuP that was measured for seven consecutive days. The results confirmed the long-term stability of the prepared nanozyme. As depicted in Figure 1F, the UV–vis spectra showed that CuP had a broad absorption band in the 1,000–1,100 nm range. The absorption spectrum of CuP irradiated with a 1064-nm laser did not change significantly, which indicates that CuP has excellent photothermal conversion stability. Transition metals with multivalent states, such as Cu and Co, have been proven to exhibit catalytic activity in tumor-specific therapy, thus destroying the redox homeostasis in the TME (Ren et al., 2020). To confirm that CuP displayed POD-like enzymatic activity, methylene blue (MB) was used as a probe for the preliminary evaluation of the degradation ability of CuP for MB at different temperatures. The results demonstrated that the POD-like activity of a given concentration of CuP increased with the increase in temperature. It is worth noting that when the temperature reached 45°C, the degradation rate of MB reached approximately 50% in 20 min (Figure 1G). To adapt to the internal oxidative stress of cell growth and survival, cancer cells regulate their ROS level by upregulating antioxidants such as GSH (Sang et al., 2020). Hence, GSH is highly expressed in tumor cells, which affects their sensitivity to RT to a certain extent. DTNB was used as a probe to test the GSH–OXD-like activity of CuP. The findings suggested that the consumption of GSH by CuP increased with time, which could be attributed to the redox reaction between GSH and the active site of Cu^2+^ (Figure 1H).

**FIGURE 2:**
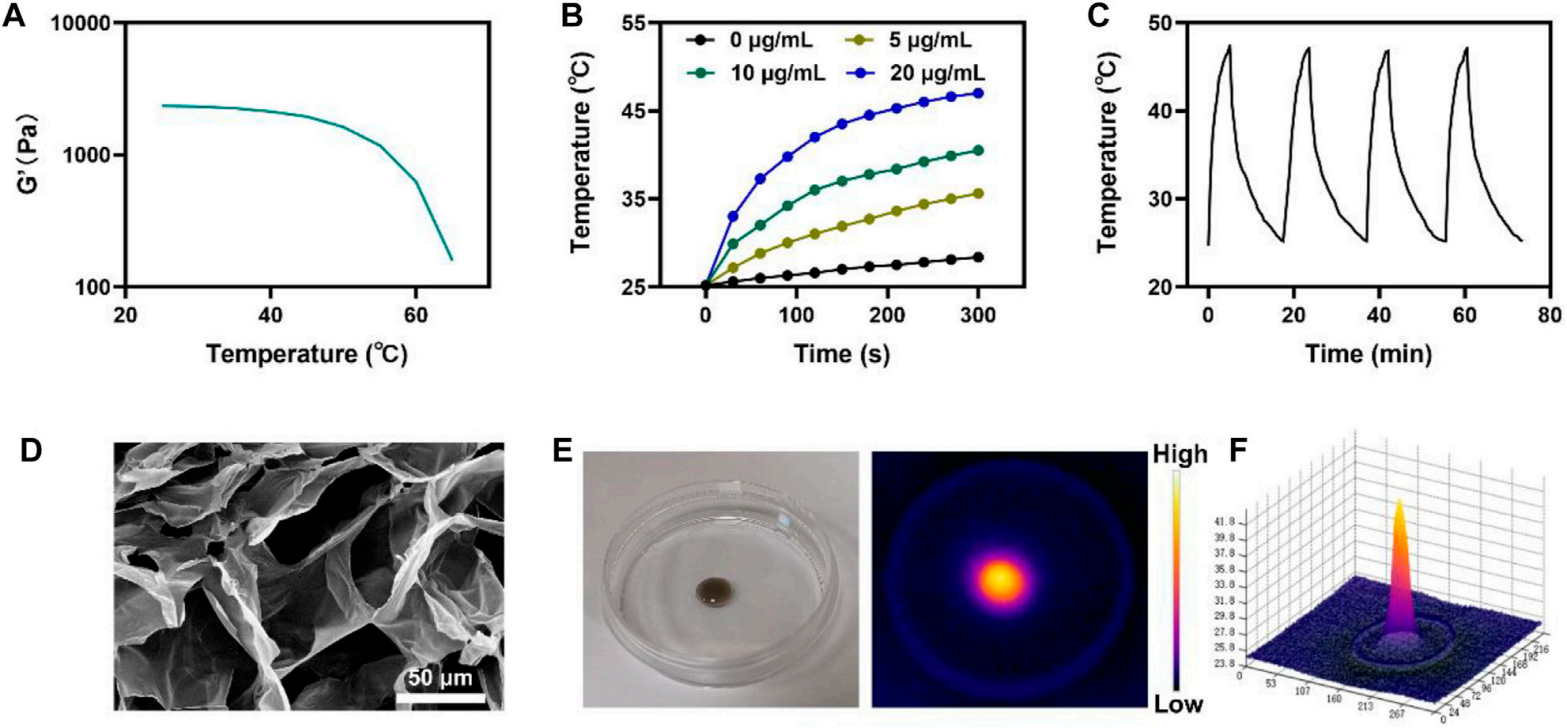
**(A)** Temperature-dependent Rheological curves for the prepared CH **(B)** Temperature changes of CuP NPs at various concentrations under a 5 min irradiation from a 1,064 nm laser at 0.5 W/cm^2^ **(C)** Heating curve of CH for four cycles having a 0.5 W/cm^2^ power intensity under irradiation by 1,064 nm laser **(D)** SEM image of the CH **(E)** The photographic pictures represent the morphology of the prepared CH and IR image during laser irradiation **(F)** Relevant 3D temperature diagram in 2 E.

For further experiments, injectable hydrogels were prepared by mixing CuP and agarose solutions as vehicles for enhancing PTT/RT. The rheological properties of CH were examined at different temperatures. The results showed that the hydrogel gradually softened with the increase in temperature and the storage modulus continued to decrease, thus leading to the controlled release of CuP in the hydrogel (Figure 2A). When the temperature and injection time were controlled, CH gelled rapidly *in situ* in the tumor. Owing to the excellent optical absorption of CuP in the NIR II region and its ability to respond to the conversion of light energy into heat energy, the photothermal heating capacities of CuP and CH were evaluated comprehensively using the photothermal imaging system. Figure 2B portrays the heating effect of CuP at different concentrations under the irradiation of a 1064-nm laser (0.5 W/cm^2^). The temperature of the aqueous solution of CuP (20 μg/mL) increased gradually under irradiation for 5 min and finally reached 46 °C (Figure 2B). In contrast, the temperature of the deionized aqueous solution did not increase perceptibly. This finding signifies that CuP, a photothermal agent, plays a key role in photothermal stability under laser irradiation. Furthermore, according to the temperature changes in the four heating–cooling cycles of 1064-nm laser irradiation with a power of 0.5 W/cm^2^ for 5 min (Figure 2C), the heating effect remained mostly unchanged until the end of the cycle. This result indicates that CuP has good photothermal stability. Therefore, the prepared CuP displays good photothermal conversion ability and photothermal stability under the irradiation of a 1064-nm laser, thus denoting its great potential in the PTT application. Scanning electron microscope (SEM) revealed the three-dimensional network structure of the hydrogel. The complex pore structure can accommodate more carriers (Figure 2D). Hence, the photothermal heating ability of CH was tested. CH was dropped at the center of a Petri dish and irradiated with a 1064-nm laser (Figure 2E). The infrared thermogram and 3D thermogram showed that the heating effect was significant (Figure 2F). The above results demonstrate that CuP has a good heating effect under 1064-nm laser irradiation. The CuP is an excellent photothermal agent that could be used synergistically with multimodal therapy for tumors.

**FIGURE 3:**
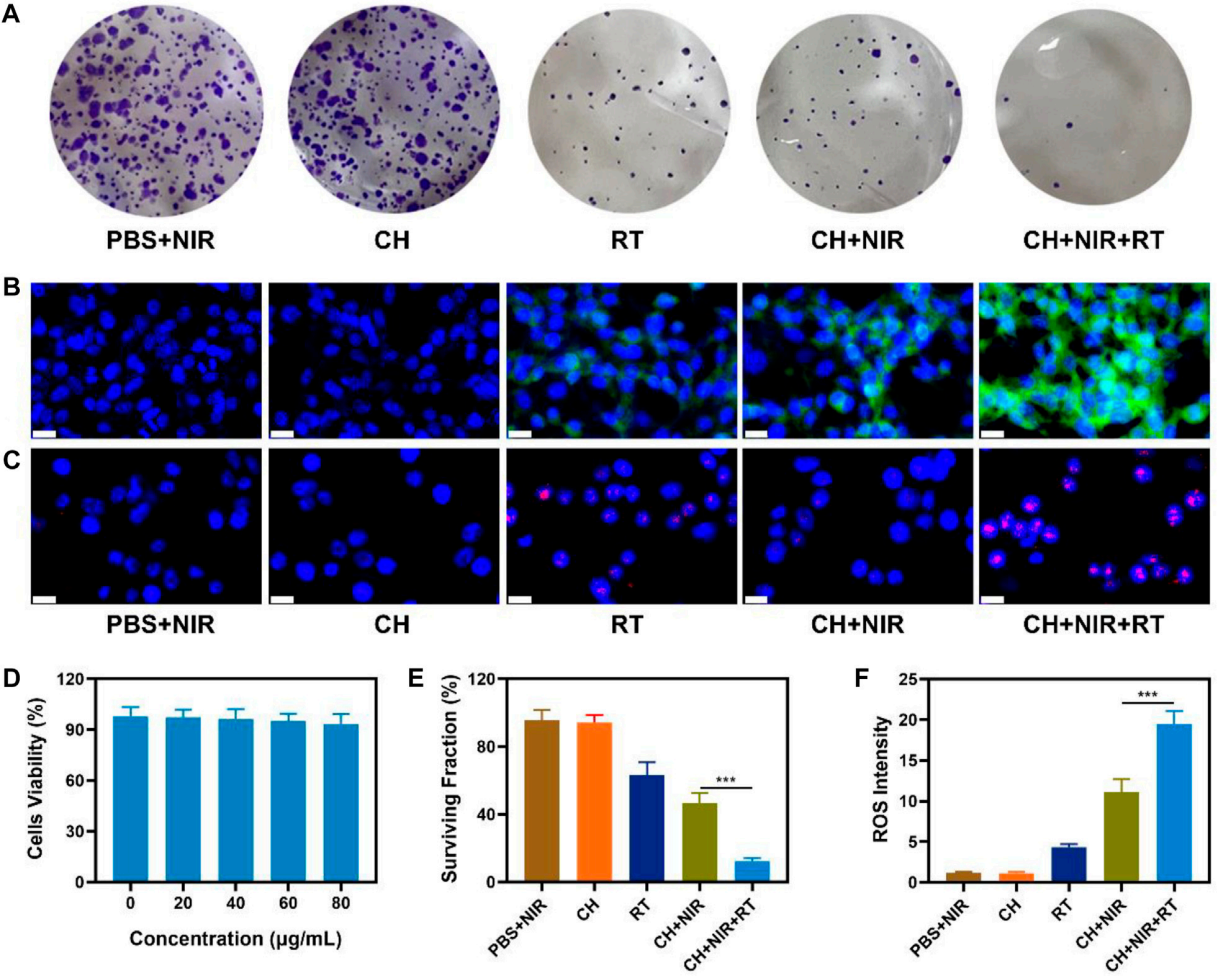
**(A)** Colony of 4T1 cells treated with different formulations and the irradiation dose was 4 Gy **(B)** CLSM images of DCFH-DA-stained 4T1 cells after treatment with different formulations. Scale bars: 20 μm **(C)** Detection of 4T1 cells’ DNA damage through immunofluorescence staining of *γ*-H_2_AX, induced by different formulations. Scale bars: 20 μm **(D)** Dark cytotoxicity of CH on 4T1 cells **(E)** *In vitro* cytotoxicity of different formulations against 4T1 cells **(F)** Corresponding quantitative analysis of ROS generation in 3A. ***p < 0.005; Student’s t-test.

In view of the aforementioned properties of CuP, the TME modulation ability and *in vitro* anticancer outcomes of CH were evaluated. The photothermal therapeutic effect and radio-sensitization ability of CuP were investigated using 4T1 cells as a model. First, the high performance of the CH synergistic multimodal treatment was confirmed using a clone formation experiment. As shown in Figure 3A, PBS + NIR and CH groups had high cell populations, whereas RT and CH + NIR groups formed relatively fewer colonies. Compared with these groups, CH synergistic NIR + RT formed fewer cell clusters and had a lower survival fraction, which was significantly different compared with CH + NIR. Studies have reported that when irradiating cells with X-rays, RT can directly damage DNA molecules in the nucleus or indirectly damage them by producing various reactive oxygen molecules with water molecules. In this study, DCFH-DA probes were used to explore the reactive oxygen species (ROS) induced by various groups. Upon entering the cells, DCFH-DA is hydrolyzed by intracellular esterase to produce DCFH, which is in turn oxidized by ROS to produce DCF that emits green fluorescence (Zhu et al., 2020). The RT group induced weak green fluorescence, as shown in Figures 3B,F. On the contrary, the CH + NIR group produced moderate green fluorescence, which could be attributed to the fact that CH subjected to laser radiation facilitates photothermal therapy by destroying the TME. CuP reduced the intracellular GSH content and converted the abundant H_2_O_2_ to produce OH, which led to increased oxidative stress and, eventually, increased ROS level. The brightest green fluorescence was achieved in the CH + NIR + RT group, which indicates that the heating effect of PTT could effectively promote the sensitization of RT to generate more ROS. X-ray induced double-strand breaks in the DNA, and H_2_AX served as the sensitivity index for monitoring double-strand breaks in the DNA. The red fluorescence in the CH + NIR + RT group was the most significant of all groups, which indicates that its nuclear DNA was most damaged (Figures 3C, Supplementary Figure S1). Furthermore, to investigate whether CH has good biocompatibility, its cytotoxicity was studied *in vitro* using 4T1 cells. MTT test results showed that even if the concentration of CuP reached 80 μg/mL (Figure 3D), it displayed low cytotoxicity toward 4T1 cells and that the cell viability remained >90%. CH-mediated PTT inhibited the repair of DNA damage after RT. CuP in the hydrogel system not only exhibited POD-like activity and produced highly toxic OH to destroy cell activity but also reduced the GSH content and made tumor cells more sensitive to ROS, thereby accentuating the radio-sensitization effect (Figure 3E). This combined strategy can augment the therapeutic efficacy by transforming non-toxic CH into a toxic therapeutic system via light stimulation and increasing the sensitivity of tumor cells to X-rays.

**FIGURE 4:**
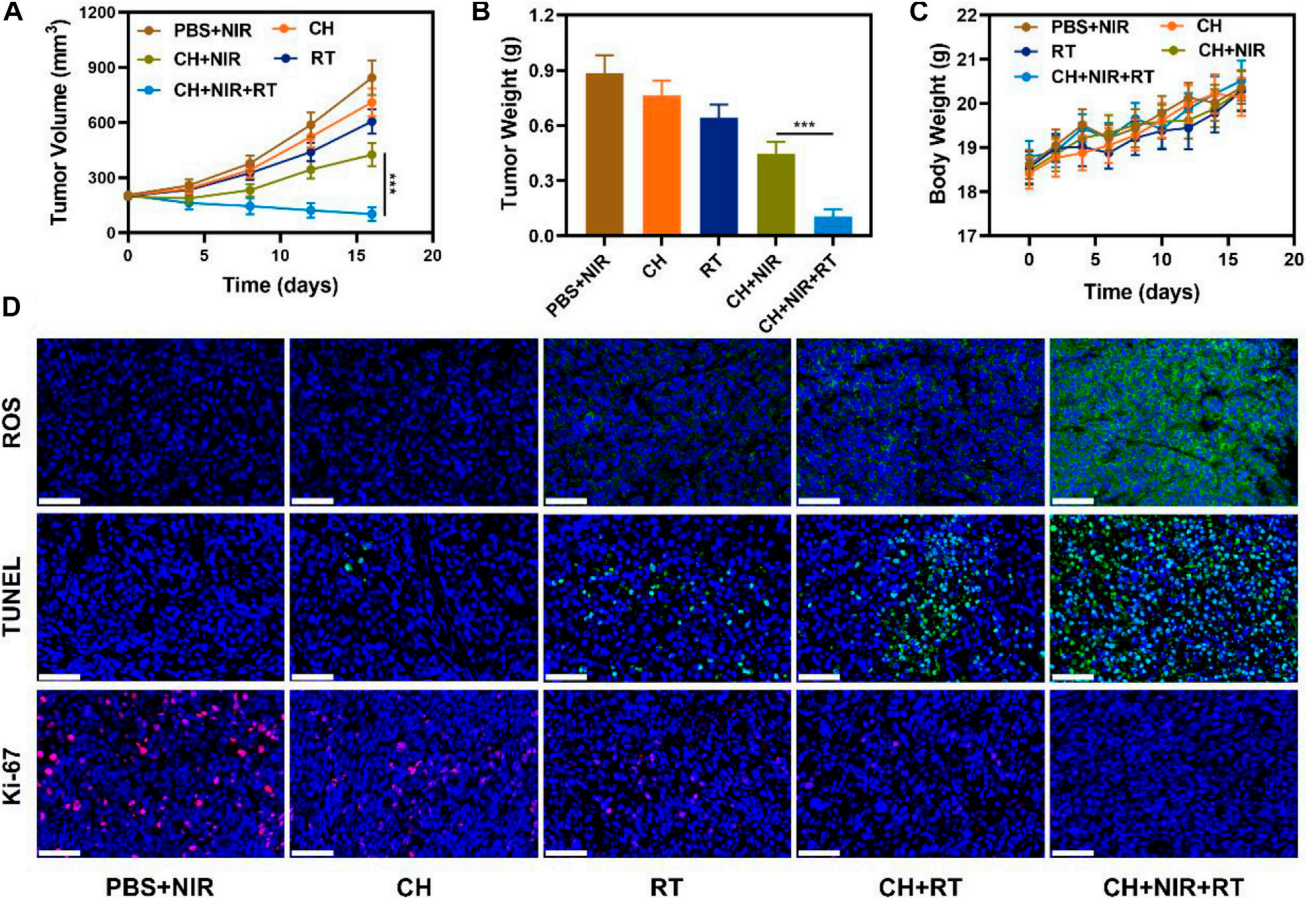
**(A)** Tumor volume changes and **(B)** tumor weights **(C)** Body weight changes of treated mice **(D)** ROS, TUNEL and Ki-67 stained tumor sections from the indicated treatment groups. Scale bars: 50 μm ***p < 0.005; Student’s t-test.

Considering the encouraging results for the validation experiments of photothermal agent and multifunctional nanozyme, the *in vivo* antitumor ability was investigated. 4T1 tumor-bearing mice were constructed via the subcutaneous injection of 4T1 cells in BALB/c mice. The mice were randomly assigned to the following five groups: 1) PBS + NIR, 2) CH, 3) RT, 4) CH + NIR, and 5) CH + NIR + RT. Tumor volumes were recorded every 4 days after providing the corresponding treatment, as shown in Figure 4A. The tumor volumes increased rapidly over time in the PBS + NIR group and were slightly inhibited by RT. On the contrary, the tumor volumes were inhibited during the first week in the CH + NIR-treated mice. Non-etheless, the treatment was not effective in the following week, and there was a rapid increase in the tumor volume. This result suggests that the combination of CH and photothermal therapy was inadequate to destroy tumor cell growth. In the CH + NIR + RT group, the tumor volume was significantly inhibited during the whole treatment cycle. CH was initially enriched in the tumor area, and after irradiation with the highly penetrating 1064-nm laser, the light energy was converted into heat energy, which destroyed the activity of tumor cells to a certain extent. Moreover, the high temperature enhanced the nanozyme activity of CuP, which converted H_2_O_2_ to produce more OH. This killed the tumor tissue and depleted GSH, the reducing substance, which made the tumor cells more sensitive to RT. After the 16th day of treatment, the mice were sacrificed and their tumors were weighed. As shown in Figures 4B, Supplementary Figure S2, the tumor weight was significantly reduced in the treatment group compared with that in the control group, which agreed with the tumor growth curve. Notable changes in body weights were not seen in any of the mice during the treatment cycle, which affirms that the regimen was not considered toxic and did not cause substantial harm to the mice (Figure 4C). To further examine the mechanism of killing the tumor cells, the tumor tissue was subjected to various staining analyses at the end of the treatment. The CH + NIR + RT group exhibited the strongest apoptotic signal and the weakest proliferative signal, together with a high content and density of ROS (Figure 4D). These findings allude that this therapeutic regimen achieved the optimal antitumor effects. To further confirm the *in vivo* safety of the system, H&E staining was performed on the main organs (heart, liver, spleen, lung, and kidney) of the mice. As illustrated in Supplementary Figure S3, compared with the PBS group, there were no significant pathological changes in the main organs of mice treated with CH + NIR + RT. This finding signifies that the treatment system has good biocompatibility and would not cause significant damage to the mice. Based on these observations, we believe that CH is a good photothermal agent and radiosensitizer, with good application potential for treating breast cancer.

## Conclusion

In this study, a Cu-doped polypyrrole-based hydrogel with tumor catalytic activity was designed for enhanced NIR-II PTT and RT. After IR stimulation, the non-toxic CH turned into a toxic treatment system. The softened CH released CuP to deplete the GSH in the TME, thereby alleviating the antioxidant capability and catalyzing the production of OH from intracellular H_2_O_2_ to kill tumor cells. The oxidative stress was thus amplified, and the approach worked concertedly with the subsequent RT to achieve a good antitumor effect. This treatment system, when combined with low-dose RT, can commendably inhibit tumor growth during the entire treatment cycle. In addition, the availability and biological safety of CH make it an extremely promising agent in clinical therapy.

## Data Availability

The original contributions presented in the study are included in the article/Supplementary Material, further inquiries can be directed to the corresponding authors.
