# Supplementary material for: Cu-doped polypyrrole hydrogel with tumor catalyst activity for NIR-II thermo-radiotherapy
Source: Front Bioeng Biotechnol. 2023 Jul 7;11:1225937. doi: 10.3389/fbioe.2023.1225937 (PMC10361615; doi:10.3389/fbioe.2023.1225937)
Supplement: Supplementary file 1 [file Table1.docx]

**Materials and reagents**

2′,7′-Dichlorofluorescin diacetate (DCFH-DA) was purchased from Solarbio Life Science (China). Pyrrole (99 %), pyrrole-3-carboxylic acid, copper chloride (II), poly (vinyl alcohol) 0588 low-viscosity (PVA-205), Methylene blue (MB), and 5,5′-dithiobis (2-nitrobenzoic acid) (Ellman's reagent, DTNB) were purchased from Aladdin Biochemica Technology Co., Ltd. Other reagents were purchased from Sinopharm Chemical Reagent (China) and Aladdin-Reagent (China).

**Preparation and characterization of copper-doped CuP nanozymes**

were successfully synthesized according to the authors’ previous work but using CuCl_2_ instead of FeCl_3_ as catalyst. First, PVA (30 mg) were mixed with 10 mL of deionized water and then heated at 90 °C under gentle stirring for 2 h. Afterward, the above mixture cooled naturally to room temperature and the freshly prepared CuCl_2_ aqueous solution (1 g, 10mL) was slowly added and stirred for 1 h. Subsequently, 200 μL of pyrrole monomer was added dropwise and the mixture was stirred for another 18 h at room temperature. The resulting CuP were collected by centrifugating and washing with deionized water repeatedly, and finally dispersed in deionized water for future use. The morphology structures of CuP were detected by the TEM (JEOL-2100). UV-vis spectra of samples were recorded by the UV-vis spectrophotometry Lambda 35 (Perkin-Elmer). The zeta potential and diameter of nanoparticles were measured by dynamic light scattering (DLS, Nano-Zen 3600, Malvern Instruments, UK). High-angle annular dark-field scanning transmission electron microscopy (HAADF-STEM) images and corresponding energy-dispersive spectroscopy (EDS) mapping analyses were acquired with a field-emission TEM (JEM-F200, Japan). The copper content was measured by ICP–AES.

**Hydroxyl radical (•OH) Generation of CuP**

Hydroxyl radical (•OH) Generation Detection of CuP. Methylene Blue (MB) was used as the •OH monitoring agent. In the experiments, 300 μL of MB solution was added to 2 mL of CuP (0.1mg/mL) and 5 μL of H_2_O_2_ (10 mM). The absorption of MB at 664 nm was recorded at various irradiation times to obtain the decay rate. Subsequently, the experiment was repeated at different temperatures.

**In vitro GSH depletion**

GSH (1 mM) was mixed with CuPPy (50 μg/mL) at 37 °C. At different time points (0, 10, 20, 40, and 60 min), 150 μL of the mixture was added into 1mL of PBS, and 7.5 μL of DTNB (10 mg/mL) was then added. The absorbance spectra of the solutions were measured by UV-vis spectroscopy.

**Preparation of CH hydrogel**

The general protocol for the hydrogel preparation is as follows. The prepared CuP (0.5mg) was added into 10mL 2% agarose solution to form CH. Then freeze dry 5 mL CH, and then conduct Scanning electron microscopy (SEM) test. SEM images were captured on a Hitachi FE-SEM S4800 instrument with an acceleration voltage of 3 kV. Rheology experiments were performed on HAAKE MARS60 (Thermo Fisher Scientific).

**Photothermal effect**

A 1064 nm NIR laser (Changchun New Industries Tech.Co., Ltd., Changchun, China) with irradiation powers of 0.5 W/cm^2^ was used to stimulate the different concentration of CuP in aqueous solution. The photothermal curve and images during laser irradiation were recorded every 30 s using an infrared camera (Fotric 225). The photothermal effect of CH under tthe NIR laser irradiation was evaluated in the same way.

**Cell culture**

4T1 cell line were obtained from the Cell Bank of the Chinese Academy of Sciences and incubated in RPMI-1640 medium supplemented with 10% FBS in a humidified atmosphere.

**Intracellular ROS detection**

4T1 cells (1 × 10^6^ per plate) were incubated with 5 different groups: (1) PBS+NIR (1064 nm, 0.5W/cm^2^, 5 min); (2) CH; (3) RT (4Gy); (4) CH+NIR, and (5) CH+NIR+RT. The CuP concentration was 100μg/mL. Then, the ROS probe DCFH-DA was added into predetermined groups and incubated for 30 min. Subsequently, the cells were washed with PBS for five times and fixed with 4% formaldehyde for 10 min. The cells were observed by a fluorescence microscope. The fluorescence intensity was measured by the ImageJ software.

**DNA Double-Strand Breaks (γ-H2AX Immunofluorescence Analysis) in Vitro**

4T1 cells (1 × 10^6^ per plate) were incubated with 5 different groups: (1) PBS+NIR (1064 nm, 0.5W/cm^2^, 5min); (2) CH; (3) RT (4Gy); (4) CH+NIR, and (5) CH+NIR+RT. Then the cells were stained with γ-H2AX antibody and DAPI. At last, the cells were imaged by CLSM.

**Dark cytotoxicity of CH on 4T1 cells**

4T1 cells were seeded in 96-well plates at a density of 5 × 10^3^ cells per well and incubated for 24 h. Afterwards, cells were incubated with CH at different concentration for 24h. At the end of the incubation, 5 mg/mL MTT PBS solution was added, and the plate was incubated for another 4 h. Finally, the absorbance values of the cells were determined by using a microplate reader (Emax Precision, USA) at 570 nm. The background absorbance of the well plate was measured and subtracted. The cytotoxicity was calculated by dividing the optical density (OD) values of treated groups (T) by the OD values of the control (C) (T/C × 100%).

**Clonogenic survival assay**

The effect of CH on the radiosensitivity of 4T1 cells was assessed by a clonogenic assay. 500 cells per flask were seeded in 25 cm^2^ flasks and cultured in normoxia for 24 h. Flasks were treated under following conditions: (1) PBS+NIR (1064 nm, 0.5 W/cm^2^, 5min); (2) CH; (3) RT (4Gy); (4) CH+NIR, and (5) CH+NIR+RT. The CuP concentration was 100 μg/mL. To allow formation of colonies, after treatment, the cells in were continuously cultured for another 10 days. To determine the clonogenic survival rate, cultures were first fixed with paraformaldehyde, and then stained with trypan blue. Colonies with greater than 50 cells were counted under the microscope, and the survival fractions (SF) were calculated using the formula SF = colonies counted/cells seeded.

**Animal tumor models**

Female BALB/c mice aged 4-5 week were purchased from Vital River Company (Beijing, China). 100 μL of 4T1 cell suspension (1×10^6^ cells per mL) were subcutaneous injected into each mouse to construct the tumor models. The animal experiments were carried out according to the protocol approved by the Ministry of Health in People’s Republic of PR China and were approved by the Administrative Committee on Animal Research of the Wuhan University.

**Evaluation of intratumoral oxidative stress**

Female BALB/c aged 5-6 week were purchased from Vital River Company (Beijing, China). Balb/c mice were subcutaneous injected with 5 × 10^6^ 4T1 cells into the right flank. When tumors reached 300mm^3^, the mice were divided randomly into five different group (n=5): (1) PBS+NIR (1064nm, 0.5W/cm2, 5min); (2) CH; (3) RT (4Gy); (4) CH+NIR, and (5) CH+NIR+RT. The CuP dose was 10mg/kg. The irradiation was performed 0.5 h after injection. DCFH-DA was injected into tumor tissue by intratumoral injection before light exposure. The cryosections were observed by a confocal laser scanning microscope (CLSM).

**In Vivo Therapy.**

The 4T1 tumor model was used. When tumors reached 200mm^3^, tumor bearing mice were treated by X-ray irradiation (4Gy). And then the mice were divided randomly into five different group (n=5): (1) PBS+NIR (1064nm, 0.5W/cm2, 5min); (2) CH; (3) RT (4Gy); (4) CH+NIR, and (5) CH+NIR+RT. The CuP dose was 10mg/kg. The irradiation was performed 0.5 h after injection. The treatment was conducted every four days. Mice body weight and tumor volume were monitored every 4 days. After treatment, all the mice were sacrificed. Five main organs (heart, liver, spleen, lung and kidney) and tumors of all mice were harvested, washed with PBS, and fixed with paraformaldehyde for histology analysis. And the tumor tissues were weighed, and stained with TUNEL and Ki67 and finally examined by using a confocal laser scanning microscope (CLSM; IX81, Olympus, Japan).

**Statistical analysis**

Data analyses were conducted using the GraphPad Prism 5.0 software. Significance between every two groups was calculated by the student’s t-test. *P < 0.05, **P < 0.01, ***P < 0.005.


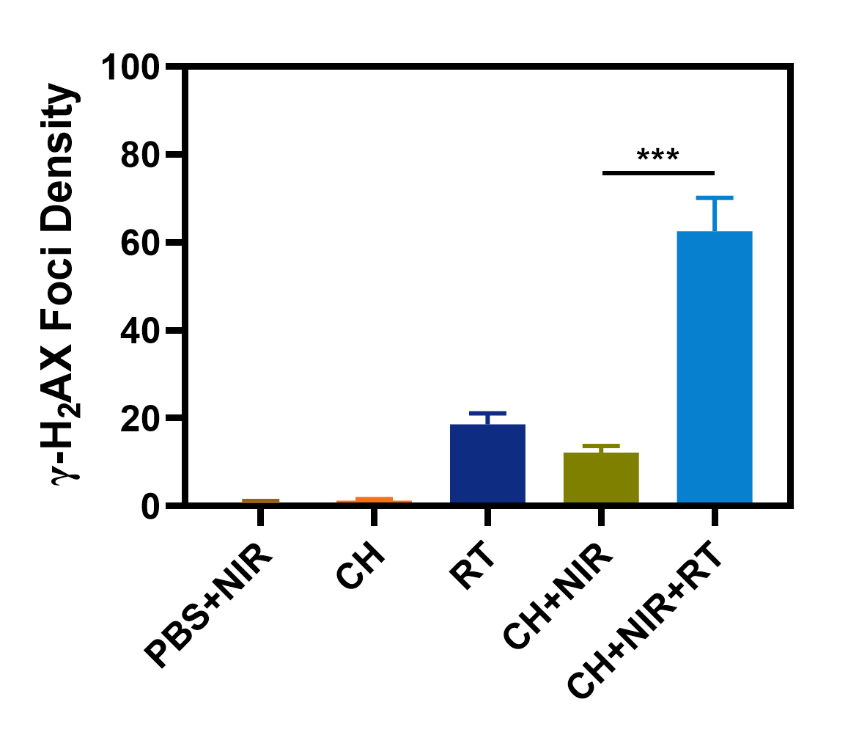


Figure S1. Quantification of γ-H2AX foci density based on

counts of at least 100 cells per treatment group.


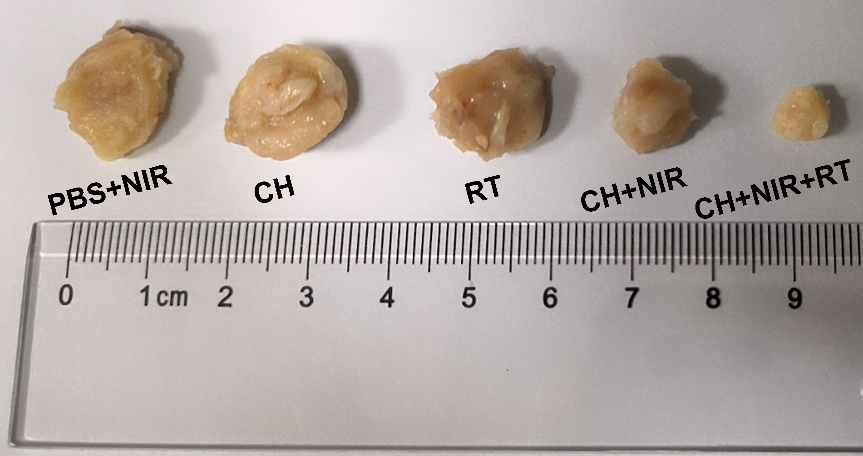


Figure S2. Typical photo images of excised tumors of different groups on the 16th day.


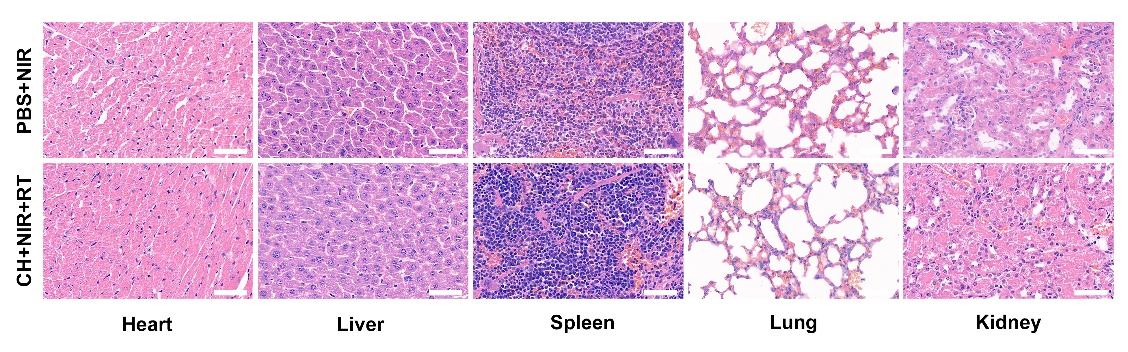


Figure S3. Histopathological analysis results (H&E stained images) of the major organs, heart, lung, liver, kidneys, and spleen, of mice that were exposed to different treatments 16 days post-injection under laser irradiation.
